# Inhibitory effect of natural compounds on quorum sensing system in *Pseudomonas aeruginosa*: a helpful promise for managing biofilm community

**DOI:** 10.3389/fphar.2024.1350391

**Published:** 2024-04-02

**Authors:** Aref Shariati, Milad Noei, Marzieh Askarinia, Amin Khoshbayan, Abbas Farahani, Zahra Chegini

**Affiliations:** ^1^ Infectious Diseases Research Center (IDRC), Arak University of Medical Sciences, Arak, Iran; ^2^ Department of Genetics, Faculty of Advanced Science and Technology, Tehran Medical Sciences, Islamic Azad University, Tehran, Iran; ^3^ Department of Microbiology, School of Medicine, Hamadan University of Medical Sciences, Hamadan, Iran; ^4^ Department of Microbiology, School of Medicine, Iran University of Medical Sciences, Tehran, Iran; ^5^ Molecular and Medicine Research Center, Khomein University of Medical Sciences, Khomein, Iran

**Keywords:** natural compounds, *P. aeruginosa*, quorum sensing, biofilm, curcumin

## Abstract

*Pseudomonas aeruginosa* biofilm is a community of bacteria that adhere to live or non-living surfaces and are encapsulated by an extracellular polymeric substance. Unlike individual planktonic cells, biofilms possess a notable inherent resistance to sanitizers and antibiotics. Overcoming this resistance is a substantial barrier in the medical and food industries. Hence, while antibiotics are ineffective in eradicating *P. aeruginosa* biofilm, scientists have explored alternate strategies, including the utilization of natural compounds as a novel treatment option. To this end, curcumin, carvacrol, thymol, eugenol, cinnamaldehyde, coumarin, catechin, terpinene-4-ol, linalool, pinene, linoleic acid, saponin, and geraniol are the major natural compounds extensively utilized for the management of the *P. aeruginosa* biofilm community. Noteworthy, the exact interaction of natural compounds and the biofilm of this bacterium is not elucidated yet; however, the interference with the quorum sensing system and the inhibition of autoinducer production in *P. aeruginosa* are the main possible mechanisms. Noteworthy, the use of different drug platforms can overcome some drawbacks of natural compounds, such as insolubility in water, limited oral bioavailability, fast metabolism, and degradation. Additionally, drug platforms can deliver different antibiofilm agents simultaneously, which enhances the antibiofilm potential of natural compounds. This article explores many facets of utilizing natural compounds to inhibit and eradicate *P. aeruginosa* biofilms. It also examines the techniques and protocols employed to enhance the effectiveness of these compounds.

## 1 Introduction

Most bacteria in nature, including those that cause hospital-acquired illnesses, exist in the form of biofilms, which are communities of microorganisms attached to surfaces (Persat et al., 2015). Biofilm communities are surrounded by a strong matrix consisting of proteins, extracellular DNA, and polysaccharides. This matrix makes them less vulnerable to antimicrobial drugs and the immune system (Walsh et al., 2019a). The concept of bacterial biofilm was initially introduced in 1987. It refers to a population of microorganisms that can attach to surfaces and produce an exopolysaccharide and extracellular matrix (Chegini et al., 2020). Biofilms readily develop on foreign objects introduced into the body, such as intravascular and urinary catheters, prosthetic valves, intrauterine devices, contact lenses, and other foreign objects, as well as on surfaces within the body (Shariati et al., 2022). Biofilms exhibit significantly higher resistance to antibiotics, ranging from 10 to 1,000 times, compared to individual planktonic cells. This enhanced resistance is mostly attributed to the limited ability of antibiotics to penetrate the intricate polysaccharide matrix, known as glycocalyx, present in biofilms (Spoering and Lewis, 2001).

*Pseudomonas aeruginosa* is a Gram-negative and rod-shaped bacterium with a pronounced tendency to form biofilms. It is also an opportunistic pathogen that exhibits multi-drug resistance (MDR) (Walsh et al., 2019a). Infection with this bacterium results in diseases characterized by a significant death rate in individuals suffering from cystic fibrosis, cancer, severe burns, and immunocompromised patients (Bahramian et al., 2019). This bacterium exhibits the ability to persist on water, various surfaces, and medical devices through the utilization of its potent adhesive elements, including flagella, pili, and biofilms. *P. aeruginosa* is highly prevalent in both natural and man-made settings, including lakes, hospitals, and domestic sink drains (Remold et al., 2011). The rising prevalence of this bacteria in generating diverse diseases and its contribution to the rise of antibiotic resistance has led to a significant global issue of treatment failure. *P. aeruginosa* exhibits substantial inherent resistance to many antibiotics, such as beta-lactams, fluoroquinolones, and aminoglycosides, leading to notable rates of morbidity and mortality (Breidenstein et al., 2011).

Therefore, the presence of biofilms and the intrinsic and acquired antibiotic resistance mechanisms of *P. aeruginosa* have led to a rise in the occurrence of MDR strains in recent years, with very few medications that are completely effective in halting the growth of this bacterium. Moreover, numerous antibacterial medications are now linked to constraints such as heightened resistance, limited antibacterial range, toxicity, and elevated treatment expenses for patients (Lone and Ahmad, 2020). To this end, scientists are seeking novel methods to impede the growth of *P. aeruginosa* biofilms (Lou et al., 2015). The utilization of natural compounds as novel and efficacious medicines has acquired a prominent position in clinical therapies (Al-khazraji et al., 2022; Alarkwazi et al., 2022; Hossain et al., 2022; Salman, 2022). Research has demonstrated that plant-derived substances, such as essential oils (EOs), extracts, and pure chemicals, have notable impacts on bacteria that are resistant to treatment and capable of producing biofilms (Ahmad et al., 2015).

The natural compounds derived from various plant components, including roots, leaves, and fruits, provide therapeutic attributes that exhibit distinct medicinal effects upon modification (Rangel et al., 2018). Plant extracts have been examined for their inherent antioxidant, antibacterial, anti-inflammatory, and antiseptic properties, as well as their role as precursors in the production of medicinal compounds (Radulović et al., 2013). In this regard, this review article will examine various aspects of the interaction between natural compounds with the quorum sensing (QS) system and the biofilm community of *P. aeruginosa*. Additionally, it will explore different drug delivery platforms that can enhance the therapeutic effectiveness of natural compounds and promote their extensive utilization in clinical practice.

## 2 Quorum sensing in *P. aeruginosa*

QS is a bacterial communication system that is dependent on cell density. It allows *P. aeruginosa* to coordinate with different determinants of virulence factors, which helps the bacteria colonize the host and develop resistance to antibiotics. QS is a system that operates on the principle of signal-response. In this system, the levels of secreted signal molecules, known as autoinducers (AIs), rise as the population density increases. These AIs are then recognized by transcriptional regulators that control specific genes (Davies et al., 1998). *P. aeruginosa* mainly possesses two QS systems: *lasI*/*lasR* and *rhlI*/*rhlR.* In these systems, *lasI* and *rhlI* (signal synthase genes) synthesize AIs like N-(3-oxododecanoyl)- L-homoserine lactone (OdDHL) and N-butyryl-L-Homoserine lactone (BHL), respectively. These AIs are then specifically recognized by intracellular receptors, LasR and RhlR. *P. aeruginosa* possesses an additional QS system known as *Pqs*, which specifically detects the *Pseudomonas* quinolone signal (PQS, 2-heptyl-3-hydroxy-4(1H)-quinolone). This system interacts with the transcriptional regulator PqsR (MvfR). Upon stimulation by their endogenous signals, these receptors will form homodimers and function as transcription factors to control the expression of the specified gene(s). Under normal circumstances, the *las* system is commonly considered the primary controller of the QS systems in *P. aeruginosa* and triggers the activation of both the *Rhl* and *Pqs* circuits (Lee and Zhang, 2015).

The DNA-binding transcription regulator LasR activates the production of the *rhl* and *pqs*, QS circuits, as well as virulence factors including *lasA* (staphylolytic), *lasB* (elastase), *toxA* (exotoxin A), *aprA* (alkaline protease), and *hcnABC* (hydrogen cyanide synthase). Therefore, under normal circumstances, the *las* system has traditionally been considered the primary controller of the QS systems *in P. aeruginosa*. Within this bacterium, the second QS pathway, known as the Rhl system, triggers the activation of the *rhlI* gene (responsible for signal synthesis), resulting in the production of BHL, which is recognized by the RhlR receptor. The BHL-RhlR complex serves as the transcriptional activator for various genes, including rpoS (a sigma factor associated with stationary phase), pqs, rhlAB (genes involved in rhamnolipid synthesis), lecA (a type I lectin), and lecB (a type II lectin) (Kim et al., 2015; Rathinam et al., 2017).

*P. aeruginosa* employs N-acyl homoserine lactone (AHL) and non-AHL-mediated cell density-dependent complicated QS signaling to control its virulence. To achieve this objective, AHL-driven QS consists of two hierarchical subsystems known as LasI/R and RhlI/R (Gambello and Iglewski, 1991; Pearson et al., 1995). The LasI/R and RhlI/R systems employ 3-oxo-C12-HSL and C4-HSL, respectively, to control the expression of approximately 10% of the genes in the *P. aeruginosa* genome. These genes control important virulence factors, including elastase, protease, hydrogen cyanide, pyocyanin, lipase, pyoverdin, rhamnolipid, swarming, and biofilm formation (Schuster et al., 2003; Wagner et al., 2003). Furthermore, *P. aeruginosa* employs two intercellular signal molecules, namely, 2-heptyl-3-hydroxy-4-quinolone (PQS) and 2-(2-hydroxyphenyl)-thiazole-4-carbaldehyde (IQS), which are not AHL-based, to control its fundamental pathogenicity (Tang et al., 1996; Pesci et al., 1999; Smith and Iglewski, 2003; Lee et al., 2013; Sethupathy et al., 2016).

Therefore, as mentioned, given the substantial role of the QS mechanism in microbial pathogenicity, inhibiting QS serves as an alternate strategy for managing nosocomial infections associated with biofilms. Anti-QS drugs, both synthetic and natural, have demonstrated efficacy in combating *P. aeruginosa* infections by reducing the thickness of biofilms and diminishing pathogenicity (Kumar et al., 2009). It is noteworthy to mention that QS suppression primarily happens through two methods: signal degradation and signal mimicking. These mechanisms lead to the inhibition of genetic control systems and the disruption of downstream virulence and biofilm genes (Ni et al., 2009; Rathinam et al., 2017). QS/biofilm inhibitors have been observed to impose limited or no selection pressure on infections to develop resistance. This is because they specifically target the production of virulence factors and the creation of biofilms without significantly affecting the growth of the pathogens. Therefore, it is crucial to identify and assess novel non-toxic substances that can effectively combat biofilm infections by inhibiting bacterial communication and biofilm formation. Various natural compounds have a considerable impact on different aspects of QS in *P. aeruginosa*. In the following part, we will explore the interactions between natural compounds and the QS system in this bacterium.

## 3 Curcumin

Curcumin is a curcuminoid compound that is present in *Curcuma longa*, a medicinal plant from the ginger family. Curcumin is widely regarded as a remarkable phytochemical due to its anti-Alzheimer, anticancer, apoptotic, anti-inflammatory, and antioxidant activities. In addition, curcumin is renowned for its antibacterial and antifungal characteristics, making it a popular ingredient in traditional medicine in India, China, and other Southeast-Asian nations. Numerous reports indicated that this remarkable phytochemical can also function as QS inhibitors, as demonstrated in studies with *Candida albicans* and *Aeromonas hydrophila* (Neyestani et al., 2019; Tanhay Mangoudehi et al., 2020). To this end, scientists are interested in employing this compound for degrading *P. aeruginosa* biofilm.

Sethupathy et al. (2016) found that curcumin reduces the activity of QS and the production of biofilms by disrupting the balance of iron levels and the response to oxidative stress in *P. aeruginosa*. This study found that curcumin modulates proteins responsible for the detoxification of reactive oxygen species (ROS), iron uptake, the tricarboxylic acid cycle, and the synthesis of pyoverdin and pyocyanin. Consequently, the authors propose that curcumin regulates the response to oxidative stress and the acquisition of iron in *P. aeruginosa* PAO1 strain by reducing the activity of antioxidant enzymes such as catalase, superoxide dismutases (SOD), peroxidase, ferroxidase, etc. This inhibition leads to the prevention of biofilm formation and the controlled production of protease, elastase, and pyocyanin through QS. Another study has identified curcumin as a QS-antagonist, which can effectively inhibit the generation of extracellular polymeric substances (EPS), biofilm, pyocyanin, and rhamnolipid, along with improving the susceptibility to antibiotics (Shukla et al., 2021). Therefore, the inhibition of QS by curcumin could inhibit the virulence factor and biofilm formation of *P. aeruginosa*.

To this end, the inhibitory effect of curcumin on QS and inhibition of the *P. aeruginosa* biofilm community were reported in other studies (Rudrappa and Bais, 2008; Packiavathy et al., 2014; Bali et al., 2019; Shukla et al., 2020; Fernandes et al., 2023). For instance, a recently published study reported that curcumin has inhibitory effects on the LuxS/AI-2 and LasI/LasR QS systems, resulting in a reduction of 33%–77% and 21%, respectively. Significantly, curcumin (at a concentration of 200 μg/mL) resulted in a 21% decrease in the generation of 3-oxo-C12-HSL by *P. aeruginosa* PA14 strain (Fernandes et al., 2023). Additionally, Rudrappa and Bais (2008) found that curcumin decreased the overall production of AHLs in PAO1 bacteria, and its supplementation resulted in a decrease in bacterial pathogenicity. Similarly, the introduction of external AHL along with curcumin reversed the harmful effects of the bacteria, resulting in the restoration of greater levels of bacterial populations. These observations suggest that curcumin may affect the QS responses in PAO1 by inhibiting the synthesis of AHL or preventing the reception of QS signals.

Noteworthy, curcumin exhibits a significant structural resemblance to AHLs. Curcumin has aromatic ring structures that consist of phenols and are linked by two α, β-unsaturated carbonyl groups. The phenolic component of curcumin imitates the lactone component of AHL. The ketone moiety present in curcumin is structurally identical to the one observed in AHLs (Packiavathy et al., 2012; Packiavathy et al., 2014). To this end, the results of an *in silico* investigation showed that curcumin can form bonds with both LasR and LuxR through distinct combinations of hydrogen bonding and hydrophobic interactions. These interactions can result in the deactivation of both proteins, enabling this plant-derived organic AHL antagonist to be categorized as a QS inhibitor (Shukla et al., 2020). In line with these results, another *in silico* study also found that the LasR protein has the highest binding affinities for curcumin (Bajire et al., 2023).

Therefore, it could be concluded that the spontaneous interaction between curcumin and AHL receptors can modify their structure, preventing them from activating a group of genes that are responsible for curcumin’s role as an inhibitor of QS (Shukla et al., 2021). Recently published study showed that the process of forming biofilms in various species is controlled by QS regulation. As previously stated, curcumin hindered the development of biofilm by disrupting the signal molecule-dependent QS mechanism. Considering the prevalence of signal-mediated QS systems, it has been discovered that disrupting the QS system could potentially hinder the generation of signal-mediated biofilms and the resulting biofilm-related illnesses. Therefore, curcumin can be utilized as an anti-pathogenic substance, specifically targeting *P. aeruginosa*, due to its ability to inhibit QS. Nevertheless, curcumin, like other natural chemicals, has restricted applicability in clinical settings due to several disadvantages, including insolubility in water, limited oral bioavailability, fast metabolism, and degradation, as well as inadequate absorption from the gut and low blood plasma levels (Shariati et al., 2019).

In this regard, researchers have been interested in using nano-curcumin and various drug delivery platforms for enhancing curcumin-antibiofilm efficacy. Notably, the higher solubility and longer half-life of nano-curcumin compared to curcumin and its higher resistance to enzymatic hydrolysis have been reported in recently published studies. Nano-curcumin exhibits more tissue reabsorption and a prolonged half-life compared to curcumin. Consequently, this molecule has been extensively studied for its potential impact on biofilm formation. The bioavailability and solubility of nano-curcumin are superior to those of curcumin. This improved water solubility can be attributed to the greater surface area of nano-curcumin, which facilitates dissolution (Shariati et al., 2019; Sharifian et al., 2020; Rahimi et al., 2023). To this end, different methods, such as the wet-milling technique and the ultrasonic homogenizer sonication method, were used for the production of nano-curcumin, and it was reported that the synthesized nanoparticles could significantly inhibit *P. aeruginosa* biofilm (Shariati et al., 2019; Sharifian et al., 2020; Rahimi et al., 2023). Additionally, the combined usage of nano-curcumin and other antibacterial agents, such as natural compounds and other nanoparticles, also showed a synergistic effect against *P. aeruginosa* biofilm (Loo et al., 2016; Rahimi et al., 2023). Xue et al. (2020) also fabricated nano-curcumin using solution-enhanced dispersion by supercritical CO_2_, and the results showed better anti-P. aeruginosa activity than the original curcumin. Therefore, nano-curcumin, in comparison to the original curcumin, not only has an improved dissolution rate and bioavailability, but also has improved antibacterial activity against the *P. aeruginosa* biofilm community.

In addition to nano-curcumin, different drug platforms were also used for the enhancement of the antibiofilm activity of curcumin. In one study that was published in 2021, curcumin was loaded onto a bioactive bone substitute (hydroxyapatite, HA) using a single-step precipitation process. The release of curcumin from HA was limited, indicating the presence of robust hydrogen bonding and van der Waals contacts between curcumin molecules and calcium ions of HA, which impeded the release of curcumin. Although the curcumin release was modest, the curcumin-functionalized HA successfully prevented bacterial cell attachment and the subsequent stages of biofilm generation in *P. aeruginosa*. The findings indicated that the quantity of curcumin released from HA was adequate to hinder the formation of biofilm and regulate the count of viable planktonic cells concurrently (Lee et al., 2021). Furthermore, other studies also used curcumin-based drug platforms for inhibition of *P. aeruginosa* biofilm (Table 1). Nanocarriers and drug platforms enhance the solubility and transportation of curcumin in bacterial suspensions, hence intensifying its antibacterial efficacy. Moreover, the utilization of a drug platform can result in a prolonged and controlled release of curcumin, reducing the required dosage of this chemical and improving its therapeutic efficacy.

**TABLE 1:** The interaction of different natural compounds with *Pseudomonas aeruginosa* biofilm.

Publication year (Reference)	Natural compound	Natural compound origin	Outcome
Bose et al. (2020a)	Terpinen-4-ol	Sigma-Aldrich	Inhibition of QS and downregulated QS genes
Noumi et al. (2018)	Terpinen-4-ol	The tea tree oil	Inhibition of swarming motility
Wira Septama et al. (2023)	Terpinen-4-ol, sabinene, γ-terpinene	*Zingiber cassumunar* EOs	Significant inhibition of biofilm formation and QS system, as well as the reduction in the levels of pyocyanin, pyoverdin, and proteolytic activity
He et al. (2022)	Linalool	Linalool emulsion	The disruption of the cellular membrane structure leads to the release of intracellular substances and effectively eliminates biofilms
Pejčić et al. (2020)	Linalool, anethole α-thujone and camphor	Basil (*Ocimum basilicum*) and sage (*Salvia officinalis*)	Suppression of the growth of biofilms and decrease in the generation of pyocyanin. Both oils had a significant impact on the motility patterns of swimming, swarming, and twitching
Kačániová and Vukovic (2023)	(E)-caryophyllene	*Salvia sclarea* EO	Inhibition of biofilm formation on plastic surfaces and stainless steel
Ghannay et al. (2022b)	Cuminaldehyde, _-pinene terpinene, and p-cymene	*Cuminum cyminum* L. EO	Inhibition of elastase and protease production and flagellar motility
Kim et al. (2021)	Linoleic acid	Sigma-Aldrich	Linoleic acid shares functional and structural similarities with cis-2-decenoic acid, a diffusible signaling component of *P. aeruginosa*. Consequently, linoleic acid works as an agonist molecule in the dispersal of biofilms
Li et al. (2023)	Geraniol	Shanghai Aladdin Bio-Chem Technology	Suppressed the virulence factors by inhibiting the three QS systems
Shafiei et al. (2017)	Saponin	*Cyclamen coum*	The combination of saponin extract and ciprofloxacin resulted in the breakdown of biofilm through different mechanisms, including the reduction of stress protein production, disturbance of cell envelope integrity, promotion of motility, and perturbation of signal transduction
Chipenzi et al. (2020)	Tormentic acid congener	*Callistemon viminalis*	Detachment of the biofilm and reduction of release of capsular polysaccharides and extracellular DNA from biofilms
Gambino and Maione (2022)	Limonene	Sigma Aldrich	Limonene therapy is highly efficacious in cases where the biofilm has been cultivated under shear stress, resulting in substantial and irreversible harm to the biofilm’s structure
Luciardi et al. (2020)	D-limonene	*Citrus paradisi*	Inhibition of biofilm production, sessile viability, autoinducer production, and elastase activity
Ghannay et al. (2022a)	Carvone and limonene	Caraway	Reduced production of virulence-related factors regulated by QS systems in Gram-negative bacteria
Husain et al. (2015a)	Menthol	Peppermint oil	Strong interference with AHL regulation of virulence factors and biofilm formation
Niu and Gilbert (2004)	Cinnamaldehyde, citronellol, and eugenol	Citronellol and cinnamaldehyde were acquired from Sigma Aldrich, while eugenol was procured from Acros Organics	Cinnamaldehyde reduced biofilm formation, citronellol increased the specific biofilm formation, and eugenol did not result in a change
Chakraborty et al. (2020)	Caffeine	NR	Caffeine not only hindered the formation of biofilm but also decreased the secretion of virulence factors by *P. aeruginosa*
Husain et al. (2015b)	Caffeine	*Trigonella foenum*-*graecum* L. (Fenugreek)	Caffeine showed anti-QS potential and anti-biofilm properties
Khan et al. (2021)	Caffeine	Caffeine-loaded gold nanoparticles	The synthesized nanoparticles exhibited several properties, including the dispersal of mature biofilms, the ability to prevent biofilm formation, and to kill a variety of Gram-negative bacteria
Rosi-Marshall et al. (2013)	Caffeine	NR	Biofilm respiration was significantly suppressed by diphenhydramine, caffeine, cimetidine, and the combination therapy. These drugs, alone or in combination, affect the biofilms in streams
Norizan et al. (2013)	Caffeine	Sigma Aldrich	Caffeine was able to inhibit the production of N-acyl homoserine lactone and the swarming
Ivanov et al. (2022)	Hesperidin, hesperetin, naringenin, naringin, hesperidin, taxifolin, morin, chlorogenic acid, ferulic acid, p-coumaric acid, gallic acid, and rutin	Sigma Aldrich	Treatment with hesperetin, hesperidin, rutin, and ferulic acid resulted in a substantial decrease in biofilm formation. The therapy with naringin and taxifolin did not cause any substantial changes in biofilm formation. Hesperetin and hesperidin showed significant antibiofilm activity. Naringenin, a flavanone, exhibits greater antibiofilm efficacy in comparison to its glycoside form, naringin
Lou et al. (2015)	Tenacissoside I, chlorogenic acid, caffeic acid, p-coumaric acid, quercetin, ursolic acid, rutin, cynarin, luteolin, crocin, and benzoic acid	The burdock leaf	The primary anti-biofilm chemicals that were identified are P-coumaric acid, quercetin, caffeic acid, ursolic acid, and rutin
Danaraj et al. (2020)	4-hydroxybenzoic acid	*Seagrass Halodule pinifolia* (Miki) Hartog	The concentration of 62.5 μg/mL of 4-methoxybenzoic acid decreased the levels of proteins, lipids, and exopolysaccharides in the biofilm matrix of *P. aeruginosa*
Quatrin et al. (2017)	1-8-Cineol	*Eucalyptus Globulus*	The nanoemulsion did not show any antimicrobial activity against *P. aeruginosa*, as only 5% of the oil was present, which is ineffective against this microorganism
Kouki and Polito (2022)	p-cymene	*Eucalyptus* Species	Inhibition of biofilm ranges between 51.21% and 91.65%
O'May et al. (2012)	Epigallocathecin gallate, tannic acid, gallic acid	Sigma Aldrich	Epigallocatechin gallate and tannic acid demonstrated the ability to impede swarming movement and enhance the development of biofilms; however, gallic acid did not exhibit any impact
Ahmed and Syed (2021)	Gallo-tannin	NR	Suppression of growth and biofilm formation of the MDR *P. aeruginosa*
Taganna et al. (2011)	Tannin	*Terminalia catappa*	Inhibition of biofilm maturation
Maisetta et al. (2019)	Tannin	*Cytinus hypocistis* and *Cytinus ruber*	Cytinus extracts did not affect Gram-negative strains
Lakshmanan et al. (2019)	Tannin	*Cinnamomum tamala* leaves	This compound can affect the production of rhamnolipid and flagellin and downregulate the expression of the fliC and *rhlA* genes
Bose et al. (2020b)	α-terpineol	Sigma Aldrich	Suppressed the QS-mediated virulence factors and the production of biofilm
Chadha et al. (2023)	α-Terpineol	NR	Suppression of the biofilm formation and the synthesis of virulence factors
Kumar Bose et al. (2021)	α-terpineol	Nano-lipoidal α-terpineol	Inhibition of biofilm formation
Das et al. (2023)	Piperine	NR	Suppression of the formation of biofilm and reduction in the expression of the QS gene (*lasI*)
Pejin et al. (2015)	Phytol	NR	Reduced the biofilm formation, twitching flagella motility, and pyocyanin
Ahmad and Ansari (2022)	Fenchone	NR	Reduced the production of biofilm
Li et al. (2018)	Diallyl disulfide	Garlic oil	Inhibition of virulence factors through inactivation of genes involved in different QS systems
Häkkinen and Soković (2021)	Lactucopicrin and costunolide	*Cichorium intybus L*	Lactucopicrin and costunolide inhibited the development of *P. aeruginosa* IBRS P001 biofilms
Jagani et al. (2009)	Polyphenol, polyanacardic acid, polycardanol, ascorbic acid, catechin, epigallocatechin, tannic acid, tocopherol, anticardia acid, cardanol, salicylic acid, ethyl linoleate, and tocopherol	Sigma Aldrich	A significant reduction in biofilm formation by *P. aeruginosa* was observed with all compounds except tocopherol and ethyl linoleate
Kasthuri et al. (2022)	Carvacrol and nerol	Sigma Aldrich	The effective biofilm disruption potential
Bahari et al. (2017)	Curcumin	NR	The generation of biofilms was significantly reduced. The combination of azithromycin and curcumin at 1.4 MIC had the most potent inhibitory effect on biofilm development
Di Salle and Viscusi (2021)	Curcumin	Sigma Aldrich	Decrease in biofilm formation
Ramesh et al. (2022)	Curcumin	Arjuna Natural Extracts	Decrease in biofilm formation
Targhi et al. (2021)	Curcumin	NR	Curcumin in combination with free and encapsulated nanoparticles significantly reduced biofilm formation in strains compared to free forms of curcumin. The *arr* gene, which plays a role in biofilm formation, was significantly suppressed
Gholami et al. (2020)	Curcumin	Merck (Germany)	The presence of curcumin-metal complexes and free curcumin suppressed biofilm formation by 45%–90%, and this inhibitory effect was dependent on the concentration
Khaleghi et al. (2023)	Curcumin	Acros organic (Geel, Belgium)	Curcumin-loaded hydrogel significantly reduced biofilm formation
Prateeksha et al. (2019)	Curcumin	Sigma–Aldrich	Zinc Oxide−Curcumin nanocomposites at a concentration of 2.5 μg/mL were much more efficient in inhibiting biofilm formation by 84.5% than the Nanoparticles-Curcumin (32%) and nanoparticles-zinc oxide (15%)
Roudashti et al. (2017)	Curcumin	NR	Decrease in biofilm formation
Kart et al. (2021b)	Curcumin	Sigma Chemical	Decrease in biofilm formation
Lu et al. (2022b)	Carvacrol and thymol	Sigma–Aldrich	Eradication of *P. aeruginosa* biofilms
Ramasamy et al. (2017b)	Cinnamaldehyde	NR	Cinnamaldehyde-coated gold nanoparticles inhibited biofilm formation by *P. aeruginosa* ∼65%
Ramasamy et al. (2017a)	Cinnamaldehyde	Sigma–Aldrich	The growth of *P. aeruginosa* biofilm and cells was entirely suppressed by cinnamaldehyde-conjugated gold nanoparticles at a concentration of 0.025% v/v. The nanoparticles penetrated the EPS matrix and effectively disrupted biofilms, but neither cinnamaldehyde nor silica-coated gold nanoparticles had any effect
S'Thebe N et al. (2023)	Cinnamaldehyde	NR	Cinnamaldehyde showed a 60% inhibitory effect on biofilm formation
Zhang et al. (2018)	Coumarin	Sigma-Aldrich	Wound model and microtiter plates exhibited a substantial decrease in biofilm production
Qais et al. (2021)	Coumarin	Sigma-Aldrich	Coumarin inhibited the growth of the *P. aeruginosa* PAO1 biofilm
Gutiérrez-Barranquero et al. (2015)	Coumarin	Sigma-Aldrich	Biofilm was significantly reduced in *P. aeruginosa*
Liu and Xiao (2020)	Catechin	Shanghai Hanhong Chemical	The nanocoating consisting of catechin and rare-earth ions exhibited remarkable antibacterial and anti-adhesion properties against *P. aeruginosa*, effectively inhibiting the formation of biofilms on the surface of the material

QS, quorum sensing; AHL, acyl homoserine lactone; NR, not reported; MDR, multi-drug resistant; MIC, minimum inhibitory concentration.

It should be noted that when we use a drug platform, in addition to curcumin, other compounds can also be used along with curcumin. For instance, Sadeghi Mohammadi et al. (2022) used gentamicin- and curcumin-loaded lipid-polymer hybrid nanoparticles for inhibition of the *P. aeruginosa* biofilm community. The findings demonstrated that synthesized nanoparticles have excellent antibacterial activity compared to pure curcumin at the same concentration, due to the increased solubility of curcumin with nanoparticles. Additionally, simultaneous usage of curcumin and gentamycin enhanced antibacterial activity. The hybrid nanoparticles exhibit a notable increase in both anti-inflammatory and antibacterial effectiveness when hydrophilic gentamicin and hydrophobic curcumin are used as the drug molecules for loading. Altogether, stimuli-responsive nano-systems can enhance the delivery of drugs to specific targets with spatial and temporal control. This has the potential to be beneficial for treating biofilm-related infections using topical therapy. Curcumin should be regarded as a potential co-delivery system for dual medicines with both hydrophilic and hydrophobic properties. This approach can boost antibacterial activities and effectively cure chronic and persistent illnesses associated with biofilm formation.

Finally, it is noteworthy to mention that curcumin was used in photodynamic therapy (PDT) for the reduction of bacterial biofilm. PDT is an effective method for combating antibiotic-resistant bacteria without the need for intrusive procedures. PDT utilizes non-toxic dyes and innocuous visible light to generate ROS, which specifically induces the death of microbial cells. Curcumin was employed as a photosensitizer in recently conducted research, targeting both the planktonic and biofilm forms of *P. aeruginosa* (Abdulrahman et al., 2020; Mirzahosseinipour et al., 2020; Zangirolami et al., 2020; Ghasemi et al., 2021; Silva et al., 2023). The use of curcumin, then PDT, reduced the number of viable bacteria (Ghasemi et al., 2021). Noteworthy, curcumin, acting as a photosensitizer, can penetrate the bacterial cell. Subsequently, when exposed to light of a specific wavelength, the photosensitizer molecules become activated and transfer their energy to the nearby molecular oxygen, leading to the production of ROS. ROS can interact with crucial molecular constituents of cells, such as DNA, membrane components, lipids, and proteins, resulting in a retardation of growth and ultimately the demise of the cell (Sánchez-López et al., 2020; Ghasemi et al., 2021). Abdulrahman et al. (2020) also reported that curcumin, when exposed to 405 nm laser light, decreased the synthesis of EPS by *P. aeruginosa* biofilm, therefore inhibiting biofilm formation. Furthermore, following curcumin-mediated PDT, it was observed that the genes associated with QS were downregulated. Additionally, a higher intensity of light resulted in a significant decrease in gene expression. On the other hand, Silva et al. (2023) documented that PDT serves as an antibacterial substitute; however, the arrangement of the biofilm poses challenges for photodynamic penetration. In addition, *P. aeruginosa* is a Gram-negative bacterium that impedes the interactions between the cell wall and molecules. In this particular scenario, the application of sonodynamic therapy utilizing ultrasound waves can mechanically break the biofilm. When combined with curcumin as a sonosensitizer, this process results in the generation of ROS. However, it is important to note that the level of ROS produced is insufficient for complete elimination of the microbes. Therefore, the authors reported that sonophotodynamic inactivation, the combination of photodynamic and sonodynamic, using curcumin could be a much more efficient approach for biofilm inactivation. However, this is a preliminary study, and more confirmatory studies are needed in this field.

Collectively, the recently published studies introduce curcumin as a sensitizer that can function as a photo and sonosensitizer, leading to highly effective inactivation of the *P. aeruginosa* biofilm. Curcumin-induced antimicrobial-PDT effectively hinders the production of biofilms by generating ROS. The regulation of biofilm formation may involve the reduction of the QS system in *P. aeruginosa*, leading to the elimination of EPS. Therefore, curcumin-mediated PDT has the potential to be a viable therapeutic method for controlling *P. aeruginosa* biofilm-induced infections. Further research should be conducted to explore the effectiveness of this treatment in managing biofilm communities of drug-resistant bacteria.

## 4 Cinnamaldehyde

Cinnamaldehyde, a primary constituent of cinnamomum, accounts for approximately 65% of its composition. Its antimicrobial activity may be attributed to the presence of an acrolein group (α, β-unsaturated carbonyl moiety). Despite its potent impact on pathogen infections, cinnamaldehyde is not susceptible to typical antibiotic resistance mechanisms (Bae et al., 1992). Recently, scientists have become interested in utilizing cinnamomum and its derivative components, particularly cinnamaldehyde, not only for their antibacterial properties but also for their ability to hinder microbial biofilm formation. Cinnamaldehyde effectively eradicated *P. aeruginosa* biofilms after exhibiting its antibacterial action through several routes (Firmino et al., 2018; Ferro et al., 2019; Kosari et al., 2020).

The principal mechanism by which cinnamaldehyde inhibits the formation of *P. aeruginosa* biofilm is through the reduction of QS. Recent reviews indicate that the sub-inhibitory concentration of cinnamaldehyde, without having any bactericidal effect, resulted in the suppression of both the *las* and *rhl* systems by inhibiting the activity of the regulatory proteins LasR and RhlR. This effect not only reduced the production of extracellular virulence agents such as elastase, pyocyanin, and protease but also repressed the expression of the rhamnolipid gene and impeded biofilm formation in *P. aeruginosa* PAO1. This study did not identify the precise mechanism by which cinnamaldehyde inhibits QS; however, the authors put forward the hypothesis that this chemical functions as a QS antagonist (Ahmed et al., 2019).

To this end, the synergistic effect of cinnamaldehyde and conventional antibiotics was investigated to eradicate *P. aeruginosa* biofilm by inhibiting QS. A recent study found that cinnamaldehyde suppressed the expression of *lasB*, *rhlA*, and *pqsA*, indicating its ability to inhibit QS. The concurrent application of cinnamaldehyde and tobramycin demonstrated potent inhibitory effects on QS. In addition, the utilization of combination therapy showed an additional impact of cinnamaldehyde when administered in conjunction with tobramycin and colistin, resulting in the inhibition of PAO1 biofilm growth and the dispersion of preexisting biofilm. This effect was more pronounced compared to the use of each treatment individually (Topa et al., 2020). In line with these findings, *Brackman* et al. used a *P. aeruginosa* mutant incapable of QS (due to a disruption of both *rhlI* and *lasI*, this strain cannot produce AHL signaling molecules). In comparison to the wild type, this mutant exhibited a significantly higher susceptibility to tobramycin, thereby confirming the involvement of a functional QS system in resistance. Furthermore, although cinnamaldehyde caused the wild-type strain to become more vulnerable to tobramycin, this effect was not detected in the QS mutant strain. This suggests that the cinnamaldehyde impact is dependent on the presence of a functional QS system. Therefore, these data indicate that the combination of tobramycin and cinnamaldehyde could enhance the effectiveness of a treatment by improving the ability of tobramycin to combat infections caused by biofilms (Brackman et al., 2011).

Additionally, the combination of cinnamaldehyde and gentamycin demonstrated a synergistic effect and showed potential action against QS. This combination therapy demonstrated the ability to inhibit the generation of AHL and reduce the expression of important QS genes in *P. aeruginosa* PAO1. The molecular docking analysis demonstrated robust binding interactions between the QS receptors and cinnamaldehyde, confirming its significant potential as a QS modulator. This dual treatment effectively eliminated pre-existing biofilms and suppressed the creation of new biofilms by disrupting the production of EPS (Chadha et al., 2022). Noteworthy, the authors suggested that the synergistic interaction between the drugs can be related to their unique mechanisms of action. Cinnamaldehyde causes membrane rupture and oxidative damage, while gentamycin inhibits bacterial protein production. This renders the bacterial cell more vulnerable as the medications can simultaneously target two separate cellular areas. In addition, cinnamaldehyde efficiently inhibits the QS circuits in *P. aeruginosa* by reducing the expression of QS genes and preventing the production of AHL molecules (Shaw and Wuest, 2020; Chadha et al., 2022). The mentioned function of cinnamaldehyde was attributed to its potent interaction with the QS receptors of *P. aeruginosa*. As a result, this reduces the activity of the virulence factors and the creation of biofilms associated with the QS system, consequently decreases the pathogenesis of the bacterium. Furthermore, the addition of gentamycin increases the effectiveness of inhibiting QS and enhances the ability of cinnamaldehyde to reduce virulence (Chadha et al., 2022).

In another study, Kart et al. (2021a) reported that the combined use of cinnamaldehyde and ciprofloxacin demonstrated a greater decrease in the minimum biofilm eradication concentration compared to the use of ciprofloxacin alone. The scientists found that cinnamaldehyde hindered the process of QS and the creation of alginate. As a result, it prevented the formation of biofilms by PAO1 and enhanced the effectiveness of ciprofloxacin in combating biofilms. As a result of these findings, there is a possibility that cinnamaldehyde could enhance the effectiveness of antibiotic treatment in combination therapy by reducing QS and thereby increasing the vulnerability of bacterial biofilms to antibiotics. Nevertheless, this hypothesis has not been experimentally verified. Collectively, cinnamaldehyde is a powerful phytochemical that has been shown to effectively combat the virulence of *P. aeruginosa*. It has the potential to be used alongside conventional antibiotics to develop new intervention methods. However, further comprehensive investigations are necessary to assess its therapeutic effectiveness using *in vivo* models in order to utilize its potential for combating virulent infections in future clinical applications.

Furthermore, a recent study has revealed that cinnamaldehyde not only inhibits QS but also inhibits the activity of Cyclic di-guanosine monophosphate (c-di-GMP) (Topa et al., 2018). C-di-GMP is recognized as a crucial intracellular signal and secondary messenger that regulates virulence, cell cycle progression, movement, and various other activities, including the life cycle of biofilms in multiple bacterial species (Ryan et al., 2006). To this end, Topa et al. (2018) reported that cinnamaldehyde decreased c-di-GMP expression by 66.2% after 5 h in comparison to the untreated control. To this end, cinnamaldehyde interfered with the transmembrane potential, pre-existing biofilms, and swarming motility of PAO1. The authors proposed that the carbon atoms of cinnamaldehyde could potentially form bonds with nitrogen-containing components, such as proteins, in the cytoplasmic membrane. This interaction could lead to changes in the structure of the proteins and result in a loss of membrane integrity. Nevertheless, there is a scarcity of information regarding the connection between cinnamaldehyde and intracellular c-di-GMP levels. Consequently, the precise chemical mechanism via which cinnamaldehyde influences variations in c-di-GMP levels is still unknown. Therefore, although the specific mechanism by which cinnamaldehyde counteracts the QS system is not yet understood, it seems to function as both a QS and c-di-GMP antagonist. Cinnamaldehyde not only eradicates *P. aeruginosa* biofilm but also eliminates the bacterium’s virulence factors via blocking QS-related factors and c-di-GMP. This occurrence would enhance the effectiveness of the host’s innate immunity and other antibiofilm agents. The administration of cinnamaldehyde to boost antibiofilm agents has promising potential for future research. However, there is limited understanding of the molecular-level effects of such synergy. Therefore, further investigation is necessary to validate the aforementioned discoveries.

## 5 Carvacrol

Carvacrol, a phenolic monoterpenoid, is a prominent component found in EOs derived from fragrant herbs like pepperwort (*Lepidium flavum*), oregano (*Origanum vulgare*), and thyme (*Thymus vulgaris*). Carvacrol is utilized as a food preservative, additive, and flavoring, as well as a fragrance in cosmetic items. Carvacrol has been found to exhibit several biological properties, such as antioxidant, anticancer, and antibacterial activity, according to recent studies (Sharifi-Rad et al., 2018; Javed et al., 2021). Carvacrol has been demonstrated to disrupt the cytoplasmic membrane by enhancing its fluidity, resulting in the release of ions, a reduction in the pH gradient across the membrane, and the suppression of Adenosine triphosphate (ATP) synthesis in bacteria (Ultee et al., 2002; Sharifi-Rad et al., 2018). Additionally, the results of recently published studies demonstrated that carvacrol could significantly inhibit the biofilm community of *P. aeruginosa* (Ceylan and Ugur, 2015; Walsh et al., 2019b; Pazarci et al., 2019; Tapia-Rodriguez et al., 2019; Walczak et al., 2021). For instance, the findings of a study indicated that carvacrol inhibited the formation of biofilms by up to 74%–88% for *P. aeruginosa* and decreased the enzyme activity of the biofilm by up to 40%–100% (Walczak et al., 2021). A biofilm eradication assay in another study also showed the potential of carvacrol for inhibition of *Staphylococcus epidermidis* and *P. aeruginosa* (Walsh et al., 2019b). In addition, *Pazarci* et al. identified carvacrol as the primary constituent of *Mentha longifolia* EO. This EO showed efficacy against *P. aeruginosa* and fungal biofilms that develop on the surface of steel and titanium implants. Hence, the authors suggested that the use of *M. longifolia* EO could be advantageous in treating infections associated with implant biofilms (Pazarci et al., 2019).

Noteworthy, the precise mechanism of how carvacrol interacts with bacterial biofilm has not been fully understood. However, a docking analysis conducted in a study revealed that this molecule does interact with the QS system in bacteria. The study revealed that when exposed to carvacrol, *P. aeruginosa* exhibited a 60% decrease in the production of AHLs. Importantly, this reduction in AHLs did not have any impact on the viability of the cells, suggesting a decrease in the activity of the LasI synthase enzyme. Carvacrol decreased the expression of the *lasR* gene while leaving the lasI gene unaffected. Furthermore, computational docking analysis revealed that carvacrol interacts with certain amino acids located in the active site pocket of LasI and within the binding pocket of LasR. Carvacrol action led to a decrease in AHLs, perhaps impacting LasI activity, resulting in the suppression of the sequence of signals, pathways, and virulence-related gene expression in *P. aeruginosa* (Tapia-Rodriguez et al., 2019). Therefore, the aforementioned research offers pertinent data regarding the impact of carvacrol on *P. aeruginosa* biofilm by disrupting the QS system. These discoveries can aid in the advancement of natural anti-QS compounds, which can impact the biofilm community and pathogenicity of *P. aeruginosa*.

Noteworthy, researchers also explored the synergistic effects of carvacrol in combination with other antibacterial agents for the management of *P. aeruginosa* biofilm. A study published in 2023 investigated the effectiveness of two proteolytic enzymes, pepsin and trypsin, in breaking down *P. aeruginosa* biofilms. The study also explored the combination of these enzymes with carvacrol. Pepsin and trypsin, both mammalian digestive enzymes, rely on certain amino acids in the polypeptide chain to break down proteins through hydrolysis. The *P. aeruginosa* biofilm population experienced a greater reduction when treated with a combination of pepsin or trypsin along with carvacrol compared to treatment with carvacrol alone. The reduction of biofilms was much greater when sequential enzyme treatments were followed by carvacrol treatment, as opposed to employing a single enzyme followed by carvacrol. The enhanced efficiency is also apparent in the analysis of epifluorescence microscopy. It was observed that the combined treatment involving enzymes, either individually or in sequence, followed by carvacrol, demonstrated a synergistic effect. This effect resulted from the enzymes’ ability to disperse biofilms and carvacrol antimicrobial activity. As a result, the biofilms exhibited a greater reduction in terms of both biomass and viability (Mechmechani et al., 2023). It is important to mention that these two enzymes, by potentially interacting with proteins, such as outer membrane proteins in *P. aeruginosa*, can lead to structural defects in the biofilms and weaken their protective properties. This interaction may enhance the entry of carvacrol and decrease the ability of the cells to survive. Therefore, the aforementioned discoveries indicate that proteins can serve as viable targets for elimination, enabling the successful infiltration of antibiofilm agents like carvacrol to deactivate *P. aeruginosa* cells within the biofilm (Mechmechani et al., 2023).

Gobin et al. (2022) also reported that the combination of gallic acid and carvacrol exhibits a potent and synergistic effect in eliminating both mono- and dual-species mature biofilms of *Staphylococcus aureus* and *P. aeruginosa*. The antibiofilm efficacy of gallic acid may be attributed to alterations in the characteristics of the plasma membrane. Indeed, besides modifying bacterial adhesion, these alterations can result in the release of intracellular constituents. Evidence has demonstrated that gallic acid stimulates the outward flow of potassium ions (K^+^) from *P. aeruginosa*. The release of intracellular components, such as ions, can ultimately lead to cell death through various mechanisms, including changes in gene expression, disruption of cellular ion balance, and interference with cell signaling. Moreover, gallic acid possesses the ability to form complexes with Ca^2+^ and Fe^2+^, two crucial metals required for bacterial growth and certain enzyme functions (Borges et al., 2013; Sarjit et al., 2015; Gobin et al., 2022). Therefore, gallic acid has the potential to modify the activity of membrane proteins and bacterial iron uptake mechanisms, such as siderophore. Gallic acid possesses an anti-adherence property that can disrupt biofilm, enhancing the diffusion and effectiveness of carvacrol, which in turn permeabilize the bacterial membrane and causes bacterial demise. Hence, the amalgamation of these molecules not only produces an additive effect but also enhances the activity of active molecules, resulting in the synergistic eradication properties of the combination (Gobin et al., 2022).

Another investigation also revealed that the concurrent use of carvacrol and nerol can effectively dismantle the preexisting biofilm structure and enhance the elimination of bacteria after subsequent water rinsing. Nerol is an acyclic monoterpenoid that is the cis-isomer of geraniol. It possesses a main alcohol functional group and has a more pleasant rose odor compared to geraniol (Kasthuri et al., 2022). The exceptional chemical composition of carvacrol and nerol is responsible for their extensive antibacterial efficacy across a wide range of bacteria. Carvacrol has a secondary alcohol known as phenolic alcohol, which is a–OH functional group in the benzene ring. Acyclic monoterpenoid nerol contains the functional group–OH at an acyclic ring structure called primary alcohol (Elsharif and Buettner, 2018). To this end, efficient broad-spectrum antibacterial action is achieved by combining the bactericidal effect of both carvacrol and nerol, which are monocyclic phenols and acyclic primary alcohols, respectively. Furthermore, there is speculation that the combined action of carvacrol and nerol, namely, their–OH groups, effectively depolarizes the cell membrane by dissipating pH and protons (H^+^ and K^+^) gradients across it (Kasthuri et al., 2022). Thus, as mentioned, the combined treatment using carvacrol and other antibacterial treatments, including natural products and enzymes, shows potential as a viable strategy for eliminating *P. aeruginosa* biofilm infections. Implementing carvacrol-based combination therapy would additionally decrease the use of chemical agents, energy expenditures, and water usage required for biofilm management.

Finally, it is noteworthy to mention that different drug platforms and techniques were used for the enhancement of carvacrol activity against bacterial biofilm. Mechmechani et al. (2022b) used the spray drying method, one of the most common microencapsulation techniques, to develop microcapsules containing carvacrol.

The results showed that the minimum inhibitory concentration (MIC) of carvacrol against *P. aeruginosa*, when encapsulated, was four times lower compared to carvacrol in its free form. Additionally, encapsulated carvacrol was able to reduce biofilm below the detection limit for this bacterium after 15 min of treatment (Mechmechani et al., 2022b). In line with these results, in another study, spray drying method was also used to produce novel microencapsulated (ME) proteases, pepsin and trypsin, and carvacrol. The findings of this study revealed that the sequential treatment in the order ME-trypsin, ME-pepsin, and ME-carvacrol resulted in more efficient biofilm removal with a maximum reduction of 5 log CFU/mL for *P. aeruginosa* (Mechmechani et al., 2022a). Notably, the pharmaceutical and food industries have extensively employed ME techniques to regulate the release of active compounds, enhance formulation stability, and conceal odors. Moreover, encapsulation aims to safeguard the active chemicals against external elements like light, water, oxygen, pH, etc., thereby preserving their antibacterial efficacy for an extended duration. This approach can hinder the interactions between antimicrobials and the biofilm EPS matrix, resulting in the rejection or retention of the biocide. This prevents the biocide from interacting with microbial cells, allowing for effective disinfection of the deep layers of the biofilm (Dias et al., 2015; Yan et al., 2022). Therefore, the results have demonstrated a favorable potential for utilizing ME carvacrol as a substitute for traditional sanitization approaches in combating biofilms in medical settings. However, before using these encapsulated products as anti-biofilm agents, they must undergo approval and registration by regulatory agencies.

Furthermore, in other studies, the researchers also loaded carvacrol on different materials, including chitosan polymers, antimicrobial polymers, and carvacrol-loaded polylactic acid (PLA) scaffolds. All of the mentioned platforms showed enhanced antibiofilm activity in comparison to the individual compounds. The increased effectiveness of the described drug platforms in preventing the formation of biofilms can be related to the specific transport of carvacrol to the biofilm. This transport is facilitated by the electrostatic contact between the positively charged delivery vehicles, in the form of polymeric micelles, and the negatively charged bacteria. Moreover, chitosan nanoparticles have the potential to enhance the infiltration of carvacrol into the biofilm population of *P. aeruginosa* (Namivandi-Zangeneh et al., 2020; Bernal-Mercado et al., 2022; Farto-Vaamonde et al., 2022). Therefore, the successful performance of drug platforms in enhancing the antibiofilm activity of carvacrol demonstrates the potential application of these platforms as both therapeutic agents and delivery vehicles for hydrophobic compounds or drugs. These platforms can be used to specifically deliver cargo molecules to the *P. aeruginosa* biofilm.

## 6 Eugenol

Eugenol, also known as 2-methoxy-4-[2-propenyl] phenol, is a phenolic aromatic compound primarily obtained from EO found in nutmeg, clove, basil, cinnamon, and bay leaf. It belongs to a unique group of microbiocidal phenylpropanoids and has been extensively utilized as a dental analgesic for a considerable duration (Hassan et al., 2018; Wijesinghe et al., 2021a). Eugenol can be created via guaiacol allylation using allyl chloride or produced through a biotransformation process using microorganisms such as *Escherichia coli*, *Corynebacterium* spp., and *Bacillus cereus* (Abrahão et al., 2013). Eugenol has been documented to exhibit several pharmacological activities, including anti-cancer, anti-inflammatory, analgesic, anesthetic, antioxidant, and antibacterial effects (Kozam, 1977; Reddy and Lokesh, 1994; Hassan et al., 2018; Nisar et al., 2021). Noteworthy, eugenol is a lipophilic molecule and has the potential to effectively pass through the phospholipid bilayer of the cell membrane. This causes changes in the membrane’s fluidity and permeability, ultimately leading to its disruption. Additionally, published data indicates that eugenol can enhance the protein leakage of cell membranes in both Gram-positive and Gram-negative bacteria. The compromised integrity of the cell walls and subsequent cellular leakages ultimately result in the demise of the microbial cell (Marchese et al., 2017; Wijesinghe et al., 2021b).

In addition, recently published studies reported that eugenol could inhibit the biofilm community of *P. aeruginosa* and the inhibitory effect of eugenol on the QS system of this bacterium is one of the most important possible mechanisms for inhibition of biofilm (Zhou et al., 2013; Rathinam et al., 2017; Rathinam and Viswanathan, 2018; Wijesinghe et al., 2021b; Lahiri et al., 2021; Shariff et al., 2022; Wang et al., 2023). Wang et al. (2023) reported that eugenol can reduce the amount of the QS signaling molecule 3-oxo-C12-homoseine lactone outside of cells by influencing the activity of the efflux pump MexAB-OprM. This indirectly disrupted the bacterial QS mechanism, thereby impeding the production of biofilms. The result of another study also showed that eugenol had a significant impact on the biofilm structure by causing a decrease in the protein and carbohydrate levels of the EPS. In addition, eugenol has the potential to impact the production of QS proteins, specifically LasA and LasB, as well as virulence factors like pyocyanin and rhamnolipid, which significantly impede the formation of biofilm (Lahiri et al., 2021).

In line with these findings, another investigation also reported that eugenol effectively decreased biofilm formation on urinary catheters and inhibited the production of virulence factors such as extracellular polysaccharides, rhamnolipid, elastase, protease, pyoverdin, and pyocyanin. Furthermore, computational docking analysis demonstrated a consistent molecular interaction between eugenol and LasR and RhlR receptors, indicating a strong binding affinity between eugenol and the QS receptors, as compared to the corresponding naturally released signal molecules (autoinducers). An examination of reporter strains has verified that eugenol competitively binds to a QS receptor (LasR), potentially inhibiting QS and resulting in substantial suppression of genes related to QS and virulence factors (Rathinam et al., 2017). Molecular modeling studies published in 2016 also demonstrated that eugenol, the main constituent of clove bud oil, effectively attaches to the QS receptor through hydrophobic interactions and hydrogen bonding with Arg61 and Tyr41, which are important amino acid residues of the LasR receptor (Jayalekshmi et al., 2016). Moreover, it was reported in another experiment that the inhibitory effect of eugenol on QS is likely due to its ability to decrease the ability of LasR transcription factors to detect their specific signal molecules, resulting in a decrease in the expression of quorum-controlled virulence factor genes and signal synthase genes (Rathinam and Viswanathan, 2018).

Finally, Zhou et al. (2013) data showed that eugenol inhibits the *las* system by reducing both the elastase activity of PAO1 and the transcriptional activation of *lasB* in *E. coli*. Moreover, eugenol demonstrated a decrease in both the pyocyanin production of PAO1 and the transcriptional activation of *pqsA* in *E. coli*. This suggests that eugenol acts as an inhibitor of the *pqs* system. By inhibiting the *las* and *pqs* systems, which control the expression of multiple genes linked to virulence, the use of eugenol would greatly reduce the virulence of *P. aeruginosa*. Altogether, according to the docking study and competitive test, it is hypothesized that the anti-QS actions are initiated by eugenol binding to the LasR receptor due to its demonstrated binding stability. Eugenol’s binding to the LasR signal receptor results in the inhibition of its role as a transcriptional activator. Moreover, eugenol most likely inhibited QS by reducing the activity of QS systems such as *las*, *rhl*, and pqs. Eugenol not only reduces the production of virulence components such as proteases, elastase, rhamnolipid, and pyocyanin in the biofilm community of *P. aeruginosa* but also significantly decreases their levels. Thus, it may be inferred that this molecule can impede the operation of various components of QS systems, namely, *las*, *rhl*, and *pqs*, at both the transcriptional and post-translational stages (Wang et al., 2023).

Prior research has indicated that eugenol is susceptible to degradation when exposed to oxygen, light, high temperatures, and humidity. Moreover, this compound exhibits a high degree of volatility, limited solubility in water, and inherent instability. Consequently, the aforementioned drawbacks have the potential to diminish the effectiveness, biological potency, and stability of eugenol (Karmakar et al., 2012; Shao et al., 2018). Therefore, given the drawbacks of eugenol in terms of its solubility, instability, and volatilization, employing various drug delivery methods could serve as a viable solution to address these issues and offer a stable, secure, and efficient antibiofilm agent for clinical application. To achieve this objective, *Rathinam* et al. applied sol-gel thin films containing eugenol onto urinary catheters and assessed the antibiofilm properties of the eugenol-coated portions against *P. aeruginosa*. The application of eugenol-coated silicone segments resulted in a decrease in the number of bacteria associated with the biofilm and effectively prevented bacterial attachment to the surface, even when a conditioning film was present. The study observed a notable decrease in the activity of genes related to virulence and biofilm formation, which confirms that silicone segments coated with eugenol have qualities that can block the expression of these genes and prevent biofilm formation (Rathinam et al., 2021). Noteworthy, silica sol-gels exhibit exceptional drug release characteristics, which may be extensively modified by manipulating the nanostructure network of the sol-gel. This modification results in a desirable therapeutic effect. Sol-gels are synthesized as thin films, coatings, and hydrogels for use in the antimicrobial and catalytic fields. They are also used for encapsulating enzymes, proteins, growth factors, and antibacterial agents (Dehghanghadikolaei et al., 2018; Rathinam et al., 2021). Therefore, sol-gel-coated silicone implants with prolonged release of eugenol could be a promising agent for managing *P. aeruginosa* biofilm; however, more confirmatory results are needed.

Another study also showed that eugenol and its nanoemulsion demonstrated that eugenol and its nanoemulsion exhibited 36% and 63% inhibition, respectively, of biofilm formation by *P. aeruginosa* at a dosage of 0.2 mg/mL. The eugenol nanoemulsion had a notable impact on the production of biofilm by this bacterium, in contrast to pure eugenol. In addition, eugenol and its nanoemulsion demonstrated the ability to decrease the activity of genes responsible for producing 3-oxo-C12-HSL and C4-HSL AHL. The impact was more prominent in the eugenol nanoemulsion compared to the pure eugenol for both synthase genes. Overall, due to its ability to hinder all the QS-mediated virulence factors and biofilm development of *P. aeruginosa*, the eugenol nanoemulsion shows promise as a new antibacterial and antibiofilm agent that targets QS, making it effective in controlling this bacterium (Lou et al., 2019).

**FIGURE 1:**
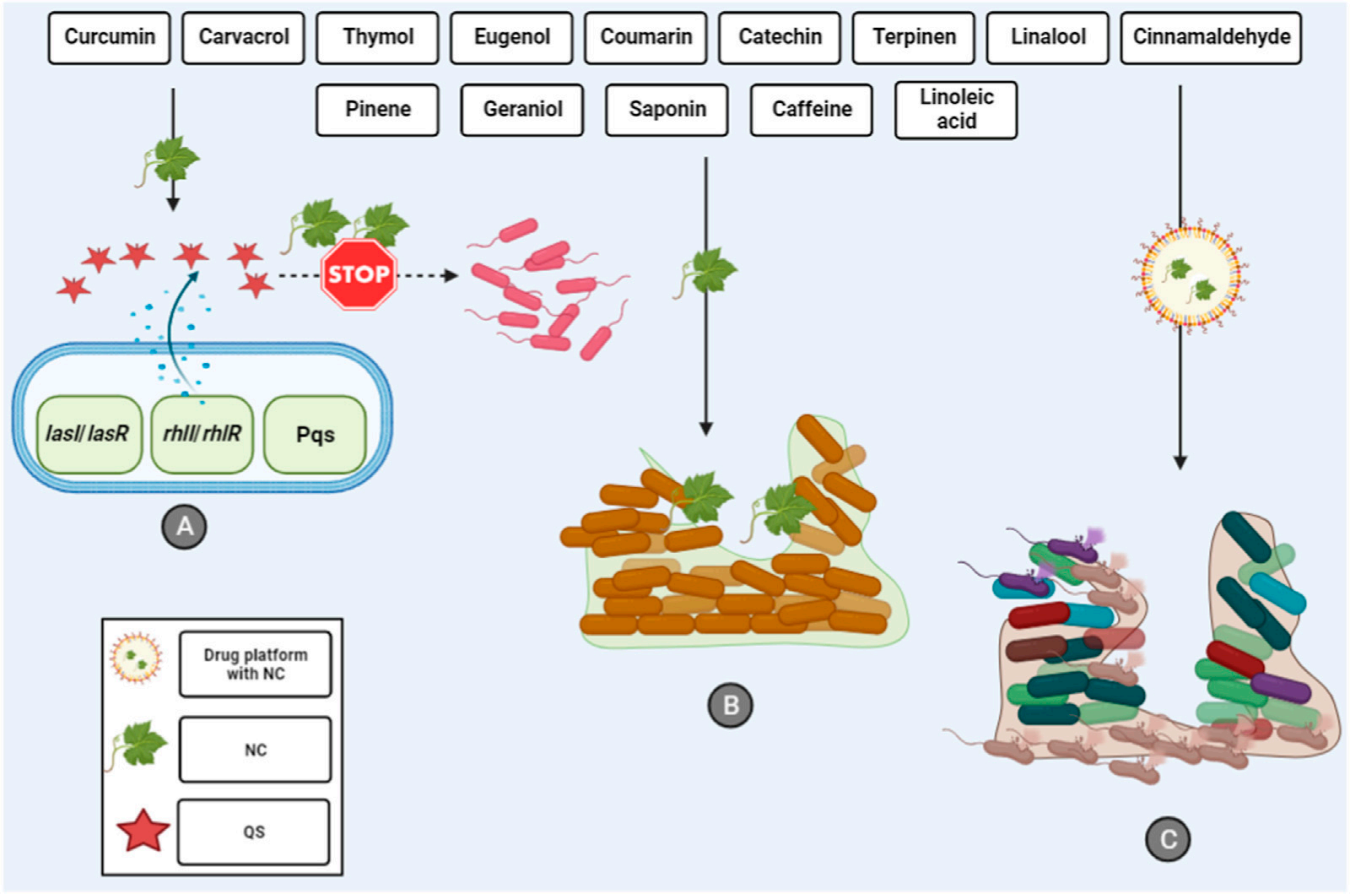
Interactions between natural compounds and *P. aeruginosa* biofilm. **(A)** Inhibition of QS system that inhibits the first step of biofilm formation. **(B)** Natural compounds can destroy the biofilm community and **(C)** The usage of different drug platforms can enhance the antibiofilm activity of natural compounds. NC, natural compounds; QS, quorum sensing.

Finally, the recently published study assessed the molecular docking of eugenol-conjugated silver nanoparticles (AgNPs) on QS regulator proteins (LasR, LasI, and MvfR). AgNPs coupled with eugenol exhibited significant binding affinities with proteins related to QS. The findings revealed that the synthesized nanoparticle can deactivate the transcriptional regulator LasR and suppress AHL synthesis. Moreover, the synthesized nanoparticle exhibits positive interactions with MvfR, potentially reducing the activity of the QS signaling system in *P. aeruginosa*. In this regard, eugenol-conjugated AgNPs may be regarded as a highly promising option for combating the production of *P. aeruginosa* biofilms (Shah et al., 2019). Therefore, as mentioned, the use of eugenol-based nanoplatforms could not only enhance the eugenol antibiofilm activity, but also suppress the QS system in *P. aeruginosa* (Figure 1). To this end, the use of these drug platforms should be considered in future studies where pure eugenol is not effective against the biofilm community.

## 7 Catechin

Catechins are bioactive compounds that are classified as polyphenolic phytochemicals. Catechins are present in a diverse range of foods and herbs, such as tea, apples, persimmons, grapes, and berries. There is a growing body of research that links the use of foods high in catechins to the prevention and treatment of chronic diseases in humans. Moreover, catechins have antioxidant properties, the ability to decrease tumor growth, and antibacterial effects (Fan et al., 2017). Noteworthy, irreversible damage to the microbial cytoplasmic membrane has been reported as the antimicrobial mechanism of catechins (Hirasawa et al., 2006). To this end, the antibiofilm activity of these compounds has also been considered by researchers.

Mombeshora et al., 2021 identified catechins as the predominant phytochemical present in *Triumfetta welwitschii*, a traditional African medicinal plant. This chemical effectively decreased both the amount of capsular polysaccharide in biofilms generated by *P. aeruginosa* and the amount of extracellular DNA in these bacterial biofilms. Noteworthy, the exact antibiofilm mechanism of catechin has not been elucidated yet; however, a recently published study indicated that catechin can disrupt QS in *P. aeruginosa*. In this concept, the study’s findings demonstrated that catechin effectively decreased the formation of biofilm, elastase, pyocyanin, and rhamnolipid, while having no impact on growth. The *in silico* analysis of this work demonstrated that catechin-7-xyloside can interact with the QS regulator LasR (Zhong et al., 2020). Another study also investigated the inhibitory effect of 19 different natural compounds on the QS system of *P. aeruginosa*. The results showed a high potency of catechin to interfere with LasR; therefore, the authors suggested catechin as a potent QS antagonist (Chaieb et al., 2022). In line with these results, the co-administration of catechin and gallic acid markedly reduced the expression of *lasI* and *lasR* in *P. aeruginosa*. The docking study revealed that catechin was successfully positioned in the ligand-binding domain of LasR. Catechin formed hydrogen bonds with Thr-115 and Ser-129 amino acid residues, which are known to play a role in the interaction with the autoinducer ligand. This supports the notion that the combination of catechin and gallic acid has a strong potential to inhibit the LasR and disrupt both QS and biofilm formation in *P. aeruginosa* (Abdel Bar et al., 2022).

Finally, Vandeputte et al. (2010) found that catechin had a notable inhibitory impact on the synthesis of pyocyanin and elastase, as well as on the formation of biofilm. Additionally, catechin affected the expression of the genes *lasB* and *rhlA*, as well as the important QS regulatory genes *lasI*, *lasR*, *rhlI*, and *rhlR*. Utilizing RhlR and LasR-based biosensors, it was observed that catechin might potentially disrupt the detection of the QS signal N-butanoyl-L-homoserine lactone by RhlR. As a result, this interference leads to a decrease in the production of QS factors. Therefore, as mentioned, the *las* system is the main QS regulator of the biofilm and the interaction of catechin with this system can significantly inhibit the biofilm community of *P. aeruginosa*. To this end, catechin may be proposed as an anti-QS agent, able to reduce the biofilm, virulence, and pathogenicity of *P. aeruginosa*.

## 8 Thymol

Thymol, also known as 2-isopropyl-5-methyl phenol, is a kind of phenolic monoterpene that has a powerful smell and can dissolve well in alcohol and other organic solvents but does not dissolve well in water (Shariati et al., 2022). Thymol is plentifully present in specific plants and has been utilized in traditional medicine for an extended period due to its diverse pharmacological characteristics (Salehi et al., 2018). Thymol has been proven to possess antibacterial and anti-inflammatory properties against microbes. This compound has been found to possess potent antibacterial activities, which make it effective in treating respiratory infections, oral cavity infections, and intestinal disorders (Jo et al., 2022). Thymol has been observed to display antibacterial properties against both planktonic and biofilm communities of Gram-negative and Gram-positive bacteria such as *E*. *coli*, *S. aureus*, *Salmonella* spp., and *P. aeruginosa* (Flores et al., 2019).

According to Walczak et al. (2021), when dealing with the biofilm formed on surfaces made of polypropylene, polyvinyl chloride, polyethylene, and stainless steel, commonly used in medical technologies and food production, thymol reduced the amount of *P. aeruginosa* biofilm by 70%–77% after 3 days of exposure. In addition, following a 10-day exposure to thymol, the production of biofilm was significantly decreased by 80%–100%. In another study that was published in 2022, the antibiofilm efficacy of thymol against biofilms generated by ciprofloxacin-resistant *P*. *aeruginosa* (CRPA) was examined on tympanostomy tubes. Noteworthy, the placement of these devices frequently results in ear infections, with otorrhea being the predominant consequence in youngsters. The authors of this study observed significant reductions in the viability and adhesion of CRPA in response to thymol therapy, which depended on the concentration of thymol. Thymol exposure also hindered the development of CRPA biofilms. In addition, thymol was found to improve the elimination of fully matured biofilms created by CRPA and also contributed to a decrease in the rates of CRPA breakdown. Moreover, thymol was found to effectively eliminate CRPA biofilms that had developed on the surface of tympanostomy tubes. The data suggest that thymol is a powerful inhibitor of CRPA biofilms, making it a promising therapeutic drug for treating biofilm-related post-tympanostomy tube otorrhea caused by CRPA infection. It is noteworthy to mention that the authors propose that thymol antibiofilm activity may be due to its ability to disrupt the surface adherence of antibiotic-resistant strains and damage the structure of the cell membrane (Walczak et al., 2021).

In line with these results, the recently published study also demonstrated that thymol, with a concentration ranging from 52.33% to 62.46%, was the primary constituent in the thyme EOs. The strongest antibacterial and anti-biofilm activity was shown in thyme oil obtained through distillation of fresh plant material gathered at the onset of the flowering period. This oil demonstrated efficacy against many microorganisms, including *P. aeruginosa* (Bakó et al., 2023). Finally, a further study on the anti-biofilm properties of thymol found that this powerful plant compound is mostly present in log (30.28%) and stationary phase (20.89%) extracts. The log phase extracts exhibited the lowest MIC (25 mg/mL) in comparison to other developmental phases. The extracts obtained from the stationary phase demonstrated the most effective biofilm dispersal activity against *P. aeruginosa*, with a rate of 80%. The log phase extracts exhibited the most potent biofilm inhibitory effect against *P. aeruginosa* (66%). Overall, leaf extracts of *Plectranthus amboinicus* in the log phase demonstrated a multifaceted mechanism of action. They exhibited antibacterial and antibiofilm actions while also lowering the motility and hydrophobicity of *P. aeruginosa*, which are crucial components in the development of this pathogen (Sawant et al., 2022). Therefore, as mentioned, thymol has the potential to inhibit bacterial biofilm. Thymol’s antibacterial effectiveness is linked to its ability to disturb both the outer and inner membranes. This disruption has been observed through the following indicators: a decrease in membrane potential and the release of potassium ions, ATP, and carboxyfluorescein (Gómez-Sequeda et al., 2020). In addition, thymol can inhibit extracellular polysaccharides present in the biofilm matrix (Valliammai et al., 2020). Thymol also inhibits *cdrA* which contributes to *P. aeruginosa* biofilm formation and stabilization (Jo et al., 2022). Noteworthy, CdrA serves as the cargo for the two-partner secretion system, which is encoded by the cdrAB operon. Inside the matrix, the bound CdrA-Psl creates strong and resistant bacterial aggregates that strengthen the structure of the biofilm (Cendra and Torrents, 2021). CdrA can bind to unidentified EPS molecules, which aids in the development of biofilms and enhances their stability (Reichhardt et al., 2018). To this end, the interactions of thymol and *cdrA* could inhibit the *P. aeruginosa* biofilm; however, data in this field is limited, and more confirmatory studies are needed.

Finally*,* the results of recently published study showed that thymol, derived from the root exudates of *Sedum alfredii*, has the ability to hinder the production of protease and elastase in *P. aeruginosa*. This is achieved by reducing the expression of *lasB*, without causing any notable impact on the primary *las* system. Thymol shows promise as a potential natural inhibitor of QS (Zhu et al., 2021). Additionally, thymol showed inhibitory effects against the QS system in other microorganisms; however, data about the interactions of this compound with the *P. aeruginosa* QS system is limited. Thus, the inhibitory effect of thymol on the QS system of *P. aeruginosa* is one possible antibiofilm effect of this natural compound; however, more confirmatory studies are required on this issue (Cáceres et al., 2020; Scandorieiro et al., 2023).

Although thymol has beneficial antimicrobial effects, it is limited by poor solubility in water, easy degradation, inadequate distribution on target sites, and chemical and biological instability caused by its volatile nature. These limitations restrict the applicability of thymol as an antimicrobial agent (Piri-Gharaghie et al., 2022). In this regard, as mentioned earlier, researchers are looking for more effective ways, such as new methods of drug delivery with this compound. To this end, *Velázquez-Carriles* et al. developed a novel nanohybrid structure consisting of zinc-layered hydroxide salt (ZnLHS) and thymol to inhibit bacterial growth. The synthesized pharmacological platform effectively suppressed *P. aeruginosa* biofilm by over 90%. Noteworthy, common disinfectants induce oxidation of the cell membrane before the formation of biofilm; therefore, the authors suggested that the hydroxide salt, owing to its anionic character, can impede the synthesis of this polysaccharide (Köse and Yapar, 2017). Furthermore, scientific evidence has demonstrated that thymol effectively inhibits the expression of genes linked with biofilm development. Consequently, the combined use of these substances may enhance the rate at which biofilm formation is inhibited (Sreelatha et al., 2021). Collectively, the hydroxide salt possesses an anionic character that facilitates oxidation, whereas thymol effectively inhibits the growth of bacterial biofilm. The greater degree of inhibition found with thymol–(ZnLHS) in comparison to thymol alone can be due to the latter’s hydrophilic characteristics. The hydrophilic nature of this substance, when it is stabilized in a colloidal dispersion, can enhance its ability to spread across the polysaccharide matrix, which also has a polar nature. Unlike thymol, ZnLHS possesses a hydrophobic property that allows it to specifically engage with the bacterial membrane. Therefore, the nanohybrid improves inhibition by capitalizing on a synergistic effect (Velázquez-Carriles et al., 2022).

Furthermore, one potential approach to addressing the constraints of thymol is to encapsulate it in nanocarriers (Fathi-Achachelouei et al., 2019). Nanocarriers, due to their small size and physicochemical characteristics, enhance the stability of the compound and can transport significant quantities of medicine (Heidari et al., 2018). In this regard, Piri-Gharaghie et al. also fabricated thymol-loaded chitosan nanogels. These nanogels can be used as a drug delivery system due to their low cytotoxicity, biodegradability, and biocompatibility (Pourebrahim et al., 2021). The antibacterial activity test showed that the thymol-loaded chitosan nanogel (thymol -CsNG) decreased the MIC by 4–6 times compared to free thymol. Thus, the authors propose that the increased antibacterial properties of thymol-CsNG can be attributed to the amalgamation of chitosan with the bacterial cell membrane and the specific discharge of thymol into the bacterial cell. Furthermore, thymol-CsNG may have an anti-biofilm effect by decreasing the number of microorganisms, leading to a subsequent reduction in biofilm development. One of the reasons is that the antibiotic can more effectively enter the biofilm structure because thymol-CsNG binds to the biofilm matrix, resulting in a decrease in viable counts and a reduction in the formation of biofilms. Furthermore, the thymol-CsNG compound effectively suppressed the expression of *ompA* and *pgaB* genes in bacterial strains. This suppression is likely a result of thymol interactions with transcription factors, leading to their inactivation and subsequent inhibition of biofilm gene transcription. Consequently, the expression of biofilm genes is diminished. Based on the findings, it can be inferred that thymol-CsNG has the potential to be a good candidate for improving antibacterial and anti-biofilm properties (Pourebrahim et al., 2021; Piri-Gharaghie et al., 2022).

In addition to the methods mentioned for how to use thymol, multiple research studies have examined the combined impact of thymol and blue light (BL) and how they work together. BL with a wavelength range of 400–495 nm is an emerging antibacterial method, especially effective against skin infections. Recent research has confirmed the effectiveness of BL in eliminating bacteria, regardless of their resistance profiles (Zhang et al., 2014). The precise process by which BL operates remains ambiguous. Nevertheless, there is a widespread consensus that BL triggers the activation of internal protoporphyrin-like derivatives and initiates a cascade of ROS generation. The hazardous ROS interacts with many constituents of bacteria and ultimately causes the cell to rupture. The broad-spectrum and rapid effects of ROS reduce the likelihood of bacterial resistance to different antibiotics such as beta-lactam antibiotics (Zhang et al., 2014). Similarly, Lu et al. (2021) established an antibacterial synergy between BL and thymol. This study investigates a combinatory approach to eliminate MDR *P. aeruginosa*, targeting both planktonic cells and established biofilms. The strategy successfully reduces bacterial populations by up to 7.5 log in less than 30 min. When used in combination, BL and thymol effectively sterilized acute infected or biofilm-associated wounds and successfully inhibited the spread of infection throughout the body in mice. BL and thymol induced oxidative bursts specifically in bacteria due to the presence of proporphyrin-like chemicals that are more numerous in bacteria than in mammalian cells. These compounds convert thymol, which is normally innocuous, into blue-laser sensitizers called thymoquinone and thymohydroquinone. These agents, when exposed to light, increased the generation of ROS and triggered a series of harmful effects in bacteria without causing any harm to the human tissue. The work reveals a hitherto unrecognized characteristic of thymol as a pro-photosensitizer, similar to a prodrug, which is specifically active in bacteria (Lu et al., 2021).

Again, in 2022, the researchers of the previous study investigated another synergistic effect. The study showed that the combination of BL and oregano EO effectively decreased *P. aeruginosa* planktonic cells and mature biofilms by more than 7log_10_. In contrast, the treatment with only BL or oregano EO did not result in a substantial reduction of bacteria. The findings indicate that thymol and carvacrol are the main constituents accountable for the bactericidal properties of oregano EO. The fundamental reason for the bactericidal effect of oregano EO is its ability to enhance the generation of ROS triggered by BL. This activation stimulates the endogenous tetrapyrrole macrocycles in bacteria, leading to their destruction. Importantly, this phototoxic action is limited to the bacteria and does not harm the surrounding tissues. An examination of the ingredients of oregano EO, together with microbiological tests, revealed that carvacrol and its isomer thymol are the primary phytochemicals that work together with BL to achieve synergistic killing. This research provides compelling evidence for the use of carvacrol and thymol as helpful benchmarks in evaluating and establishing the antibacterial effectiveness of different oregano EO products (Lu et al., 2022a). Therefore, as mentioned, in addition to the use of a thymol-based drug platform, the combined use of BL and thymol could be a promising antibiofilm approach for managing *P. aeruginosa* infections; however, more confirmatory studies are needed in this field.

## 9 Conclusion

The biofilm community of *P. aeruginosa* is one of the most important reasons for antibiotic resistance in this bacterium. As reported by recently published studies, natural compounds can inhibit biofilm formation and enhance the antibiofilm efficacy of conventional antibiotics and other antibacterial agents. The exact interactions between natural compounds and *P. aeruginosa* biofilm have not been elucidated yet; however, inhibition and interference with the QS system are the main possible mechanisms that have been reported until now. Additionally, the use of different nanostructures and drug platforms could overcome the disadvantages of natural compounds such as rapid metabolism and degradation, water insolubility, and low oral bioavailability. Hence, it is vital to contemplate the utilization of drug platforms in forthcoming investigations that prioritize the assessment of the antibiofilm efficacy of natural compounds. It is important to note that there is a lack of animal studies and clinical studies on this subject. Therefore, future research should focus on evaluating the effectiveness of natural compounds in preventing biofilm formation through these types of investigations. Additionally, data about some of the natural compound interactions with the biofilm community is limited. To this end, focusing on these natural compounds can lead to finding new ways to manage the *P. aeruginosa* biofilm community.
